# Retraction: Angiogenesis Impairment in Diabetes: Role of Methylglyoxal-Induced Receptor for Advanced Glycation Endproducts, Autophagy and Vascular Endothelial Growth Factor Receptor 2

**DOI:** 10.1371/journal.pone.0343105

**Published:** 2026-02-19

**Authors:** The PLOS One Editors

Following publication of this article [1], The University of Oklahoma Health Campus (OUHC) notified PLOS that an institutional investigation determined there was falsification in Figs 3 and 6-8. Specifically, OUHC reported the following findings to PLOS: There are multiple incorrectly labelled conditions and/or sample types among the immunoblot and micrograph images reported in Figures 3E, 6C, 6E, 6F, 7B, 8A, and 8B.

Additionally, PLOS noted the following concern:

- When the aspect ratio is adjusted, lanes 1, 2, and 4 in the VEGFR2 panel in Fig 6A appear similar to the corresponding lanes in the VEGFR2 panel of Fig 6C, despite representing different experimental conditions.

During follow-up, the corresponding author indicated that they stand by the findings reported in the article [1], but stated that the data underlying the panels of concern are no longer available.

In light of the concerns raised about the reliability of the reported results, and in accordance with the request of The University of Oklahoma Health Campus, the *PLOS One* Editors retract this article.

JX did not agree with the retraction and stands by the article’s findings. HL, SY, and HZ either did not respond directly or could not be reached.

## References

[1] Liu H, Yu S, Zhang H, Xu J. RETRACTED: Angiogenesis impairment in diabetes: role of methylglyoxal-induced receptor for advanced glycation endproducts, autophagy and vascular endothelial growth factor receptor 2. PLoS One. 2012;7(10):e46720. doi: 10.1371/journal.pone.0046720 23056421 PMC3463541

